# Effect of Vehicle–Bridge Coupled Vibration on the Performance of Magnesium Phosphate Cement Repair Materials

**DOI:** 10.3390/ma14247743

**Published:** 2021-12-15

**Authors:** Sijia Liu, Long Yu, Hao Han, Feng Pan, Kai Wu, Zhenghong Yang

**Affiliations:** 1 Key Laboratory of Advanced Civil Engineering Materials of Ministry of Education, Tongji University, Shanghai 201804, China; lsj489477@tongji.edu.cn (S.L.); yulong@tongji.edu.cn (L.Y.); hanhao97@tongji.edu.cn (H.H.); 2 School of Materials Science and Engineering, Tongji University, Shanghai 201804, China; 3 Shanghai Construction No. 5 (Group) Co., Ltd., Shanghai 200063, China; panfeng@scgwj.com

**Keywords:** fiber-reinforced magnesium phosphate cement (FR-MPC) composite, vehicle–bridge coupled vibration, bonding property, pore structure, interfaces

## Abstract

This study evaluates the effect of vehicle–bridge coupled vibration on the mechanical properties of fiber-reinforced magnesium phosphate cement (FR-MPC) composites and the bonding properties of repaired systems. By means of compressive and flexural bond strengths, fiber pullout, mercury intrusion porosimeter (MIP) and backscattered electron imaging (BSE) analysis, an enhanced insight was gained into the evolution of FR-MPC performance before and after vibration. Experimental results showed that the compressive strength and flexural strength of FR-MPC was increased when it was subjected to vibration. However, the effects of vibration on the flexural strength of plain magnesium phosphate cement (MPC) mortars was insignificant. The increased flexural strength of FR-MPC after vibration could be due to the high average bond strength and pull-out energy between the micro-steel fiber and the MPC matrix. Moreover, BSE analysis revealed that the interface structure between FR-MPC and an ordinary Portland cement (OPC) substrate was more compacted after vibration, which could possibly be responsible for the better bonding properties of FR-MPC. These findings are beneficial for construction project applications of FR-MPC in bridge repairing and widening.

## 1. Introduction

Under the joint effect of heavy traffic loads and harsh environmental conditions, concrete bridges face the problem of being degraded or even damaged [1,2,3,4]. Moreover, due to an increase in population and number of vehicles, many existing bridges may be widened to carry more loads and support transportation [5,6]. In order to avoid traffic jams caused by bridge closure, it is necessary to repair or widen existing bridges without interrupting traffic [7].

Under open traffic construction conditions, the vibration of old bridge decks caused by vehicles affects the newly placed repair materials of adjacent lanes [8,9,10]. A study by Jiang et al. [11] showed that coupled vibration of a vehicle and bridge can lead to propagation of numerous micro-cracks and other defects in the concrete during the setting and the hardening period. Hong et al. [7] found that coupled vibration occurring within the first hour of casting exerts a considerable adverse effect both on the compressive strength of the repair materials, and the bond strength between the repair materials and the damaged structure. Previous studies demonstrated that repaired components exposed to vibrations too early could affect the efficiency of a repair. Therefore, determining how to ensure the early performance of repair materials to minimize the negative impact of vibration on the repaired system has become a key issue.

Magnesium phosphate cement (MPC) belongs to the family of chemically bonded ceramic (CBC) materials and gains strength via an acid–base reaction between dead burnt magnesium (MgO) and soluble acid phosphates, in the presence of water [12,13,14,15]. Due to its short setting time, super-high compressive strength at an early age and favorable bonding properties, MPC is often used for fast mending of highway pavements, airport runways, bridge decks, municipal roads and other concrete structures [16,17,18]. Construction can be completed in a quarter of an hour, and the structures may return to service within a few hours to meet the needs of rapid traffic opening [19].

Due to its high crystal composition and microstructure of hardened paste, MPC shows characteristics of brittle failure under the condition of high flexural or impact loading [20,21]. In order to improve the strength, toughness and ductility, inclusion of micro-steel fibers in MPC combinations has been approved as one of the most effective solutions [22,23,24,25]. Meanwhile, the inclusion of micro-steel fibers is also able to promote the bonding between repair materials and substrates [26]. It indicates that fiber-reinforced magnesium phosphate cement (FR-MPC) composites have exhibited the potential to be used in the fields of repairing and reinforcing flexural structure components [27].

However, few attempts have been made to evaluate the effect of vehicle–bridge coupled vibration on the performance of FR-MPC composites, which are considered promising repair materials for bridge structures. For this purpose, the current study was designed to evaluate the effect of simulated bridge vibration on the performance of FR-MPC composites. Since any imposed damage is most likely to originate from the weak interface between repair materials and their substrates, the bonding properties of FR-MPC composites are also critical to a repaired system.

In order to lay a solid foundation for the application of FR-MPC composites in the repairing or widening of bridge structures, the effects of vibration on the mechanical properties of plain MPC mortars and FR-MPC composites at early age were determined. The one-way fiber pull-out test and mercury intrusion porosimeter (MIP) test were carried out to assess the toughening effect of FR-MPC composites. Moreover, the bonding interface between FR-MPC composites and the substrate prepared with Portland cement concrete was evaluated using flexural bond strength and backscattered electron imaging (BSE) analysis.

## 2. Experimental Section

### 2.1. Materials

**Figure 1:**
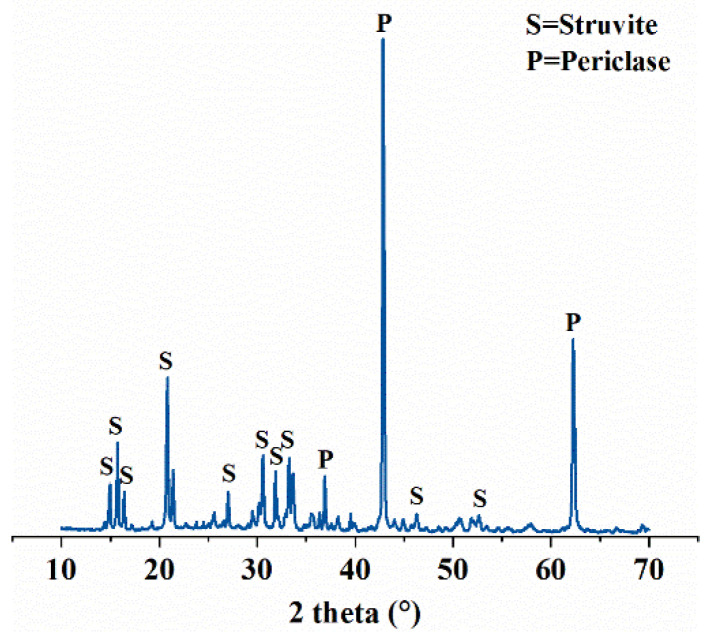
X-ray powder diffraction pattern of hardened MPC paste.

The MPC used in this work was obtained from Guizhou Magnesium Phosphate Material Co., Ltd., Guiyang, China. It was prepared from a mixture of dead burnt magnesium (MgO), ammonium dihydrogen phosphate (NH_4_H_2_PO_4_) and fly ash (FA) in a certain proportion. The chemical composition of the MPC determined through XRF analysis is shown in Table 1. Borax (Na_2_B_4_O_7_·10H_2_O) was used as the retarder and the setting time of fresh MPC paste was 22.0 min. The substrate mortars were prepared with ordinary Portland cement (P.O. 42.5) and standard sand, at a water–cement ratio of 0.5 and a sand–cement ratio of 3. Two types of micro-steel fibers were used for the preparation of FR-MPC which was acquired from Shanghai Real Strong Composite Material Co., Ltd., Shanghai, China. Detailed physical properties of the fibers are listed in Table 2. X-ray powder diffraction patterns of MPC paste powder obtained after a 3-day hydration period are illustrated in Figure 1.

### 2.2. Vehicle–Bridge Coupled Vibration Simulation

**Figure 2:**
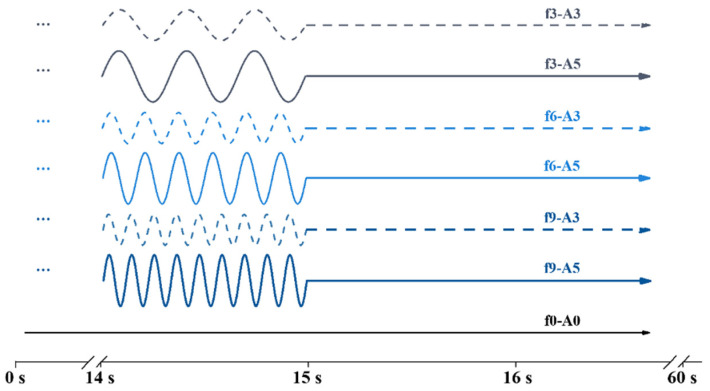
The schematic diagram of vehicle–bridge coupled vibration processes.

An electromagnetic vibration table was used to simulate the vehicle–bridge coupled vibration. The dynamic response parameters of a bridge structure caused by a dynamic load include the impact coefficient, frequency, amplitude, damping and stiffness. In this paper, the process focused on the amplitude (A) and the frequency (f) parameters. In accordance with previous studies [11,28], a vibration frequency of 0, 3, 6 or 9 Hz and a vibration amplitude of 0, 3 or 5 mm were selected. To simulate the real intermittent passing of vehicles, the vibration mode was set as: vibrating for 15 s and standing by for 45 s, as one cycle. The whole vibration program lasted 60 cycles (1 h). The specimen’s label was defined as “fX-AY”. For example, f6-A3 indicated a vibration mode of 6 Hz frequency and 3 mm amplitude. The f0-A0 specimens were prepared as the reference value: without vibration. The detailed vibration parameters and the detailed vibration modes in a single cycle are summarized in Figure 2.

### 2.3. Specimen Preparation

The mixture proportions of plain MPC and FR-MPC specimens were prepared as follows: m(MPC):m(sand):m(water) = 1.0:1.0:0.15. Compared with plain MPC, FR-MPC contained 1.0% micro-steel fibers, with a length of 7 mm, by total weight of mixture. This length and the micro-steel fibers content were adopted after considering the workability and the mechanical properties of FR-MPC. The MPC, standard sand and water were firstly mixed in a Hobart mixer and stirred continuously until a uniform mixture was obtained. Subsequently, micro-steel fibers were gradually added within 60 s and further mixing for 90 s was performed. Fresh mixtures were quickly poured into 40 × 40 × 160 mm^3^ molds, the “8-shaped” molds and the molds with half ordinary Portland cement (OPC)substrate. After casting the repair materials, vibration was performed immediately under the above-mentioned program. The mortars were demolded after the vibration and stored at constant temperature of 20 ± 2 °C and a relative humidity of 60 ± 5%, until the designated testing age.

**Figure 3:**
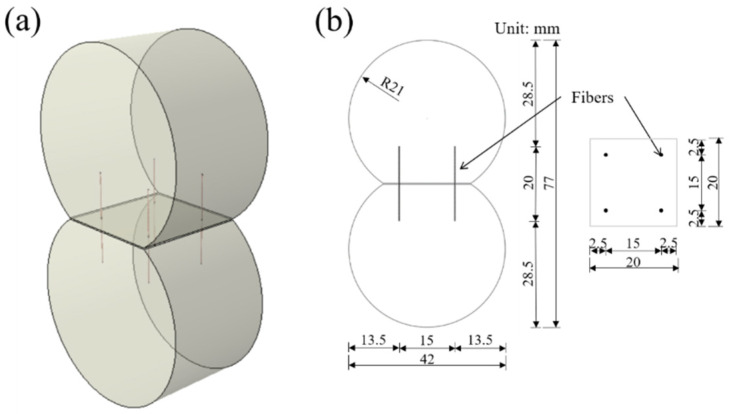
Geometrical details of “8-shaped” specimen: (**a**) 3D schematic diagram; (**b**) geometrical parameters.

The “8-shaped” specimens as shown in Figure 3a were prepared for the fiber pull-out test. Four micro-steel fibers with a length of 20 mm were inserted into the holes fixed with a plastic plate, then one side of the mold was cast with MPC as the fixed side. The embedment length was half of the fiber length. The other side was cast with MPC as the pull-out side after 1 h of hardening for the fixed side. Geometrical parameters of “8-shaped” specimen can be summarized in Figure 3b.

**Figure 4:**
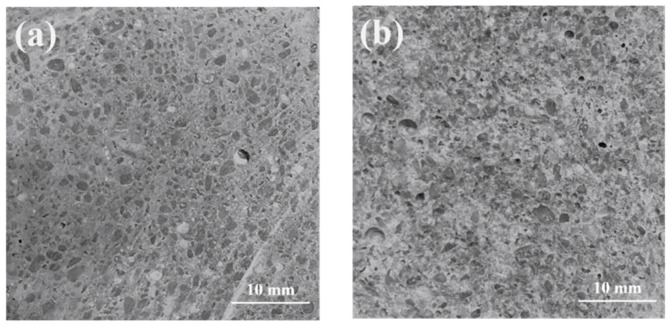
Interface roughness of ordinary Portland cement (OPC) substrate: (**a**) smooth surface; (**b**) roughened surface.

The flexural bond strength of the repaired specimen was evaluated by pouring the FR-MPC on the OPC mortar-prepared substrate. The OPC mortars were demolded after 1 day of casting and cured in standard conditions with temperature of 20 ± 2 °C and relative humidity of 95%, for an extra 27 days. Two types of substrate surface were involved in the evaluation, i.e., the smooth surface (as shown in Figure 4a) and the roughened surface (as shown in Figure 4b). The smooth surface was obtained by cutting OPC mortar into halves with a slicing machine. The roughened surface was obtained from this OPC mortar after three-point bending. The FR-MPC mixtures were placed besides the half prism of OPC substrate to form combined specimens. The details of testing methods and specimen dimensions are shown in the following section.

### 2.4. Testing Methods

#### 2.4.1. Compressive and Flexural Strength Test

Based on the procedure of the Chinese standard GB/T 17671, the plain MPC and FRMPC prismatic specimens with dimensions of 40 × 40 × 160 mm^3^ were prepared for flexural strength testing, with astrain rate of 50 ± 10 N/s. The flexural strength was the average of three parallel specimens. Broken specimens after the flexural strength test were used to measure the compressive strength with a loading rate of 2400 ± 200 N/s.

#### 2.4.2. Fiber Pull-Out Test

**Figure 5:**
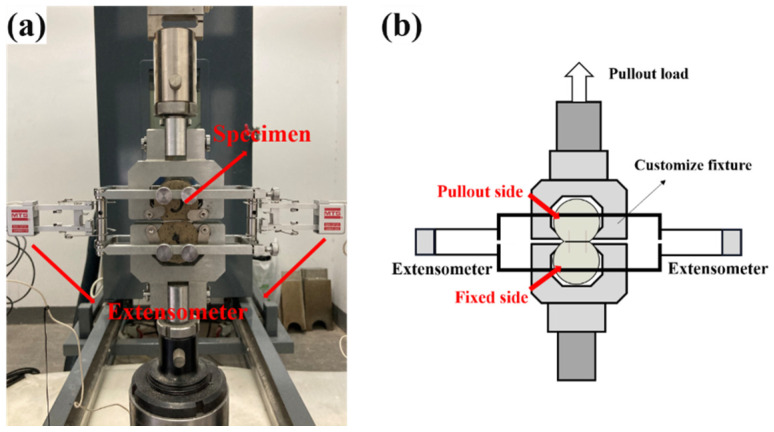
Fiber pull-out test device: (**a**) actual picture; (**b**) schematic diagram.

A fiber pull-out test was performed according to Chinese standard CECS 13. The test was conducted by using an MTS electronic universal testing machine (Shanghai, China) with a capacity of 500 N. The test device is shown in Figure 5. The “8-shaped” specimen was loaded with a rate of 0.5 mm/min. The slip between the fiber and the matrix was measured by two extensometers. Five specimens for each group were tested and averaged as the final results.

The average bond strength and pull-out energy were obtained by plotting pull-out load versus slip curve to evaluate the interface bond properties of micro-steel fiber embedded in MPC, which can be calculated using Equations (1) and (2) [29,30]:

(1) $$\tau_{av}=\frac{P_{\max}}{n\pi d_{f}l}$$

(2) $$W_{P}=\int_{0}^{2.5}P(S)dS$$

where *τ_av_* is the average bond strength in MPa, *P_max_* is the maximum pull-out load in N, *d_f_* is the fiber diameter in mm, *l* is the initial embedment length in mm, *n* is the fiber number, *W_P_* is the pull-out energy in N·mm, *S* is the fiber slip in mm and *P(S)* is the pull-out load applied at a certain slip value of *S* in N.

#### 2.4.3. Flexural Bond Strength Test

**Figure 6:**
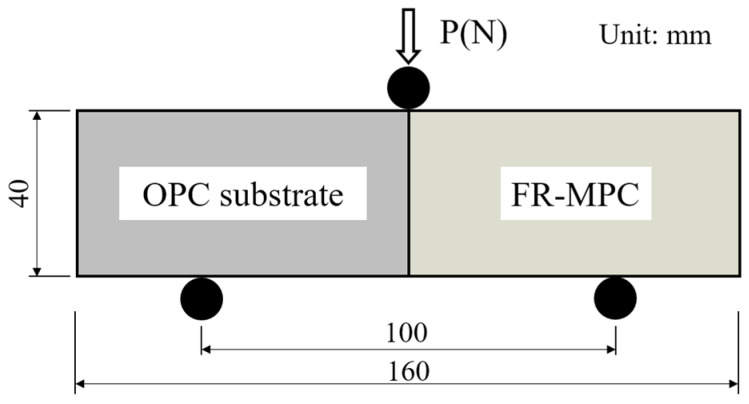
The test method for measuring flexural bond strength.

The flexural bond strength test is a direct and effective bond strength evaluation method. It is often used to evaluate the bond strength of the interface between a repair material and old OPC substrate. The dimension of the combined specimen is summarized in Figure 6. The detailed test method of flexural bond strength was the same as the flexural strength test.

#### 2.4.4. Pore Structure Evaluation

AutoPore IV 9500 mercury intrusion porosimetry (Norcross, GA, USA) was employed to determine the pore structure of MPC before and after vibration. The samples used for the MIP test were collected from the core of the “8-shaped” specimens by breaking them into bulks and immersing in ethanol to terminate hydration. The pressure range was set to 0.6895 kPa–420.595 MPa, and a contact angle of 130° was used. According to the Washburn equation, the accessible pore diameter ranged from 3.0 nm to 343.9 μm.

#### 2.4.5. Backscattered Electron Imaging Analysis

Flexural bond specimens were cut into slides across the bond interface, covering both the substrate and the FR-MPC. Samples were impregnated in a low viscosity epoxy resin, ground with 320, 500, 1200 and 2400 grit SiC paper, for 4 min each and polished with diamond paste of 3, 1 and 0.25 μm for 2 min each. Back scattered electron (BSE) images of carbon-coated samples were imaged using SEM (FEI QUANTA 200 Environmental SEM, Hillsboro, OR, USA) at an accelerating voltage of 20 kV.

## 3. Results and Discussion

### 3.1. Effect of Coupled Vibration on the Mechanical Strength of Plain Magnesium Phosphate Cement (MPC) Mortars

**Figure 7:**
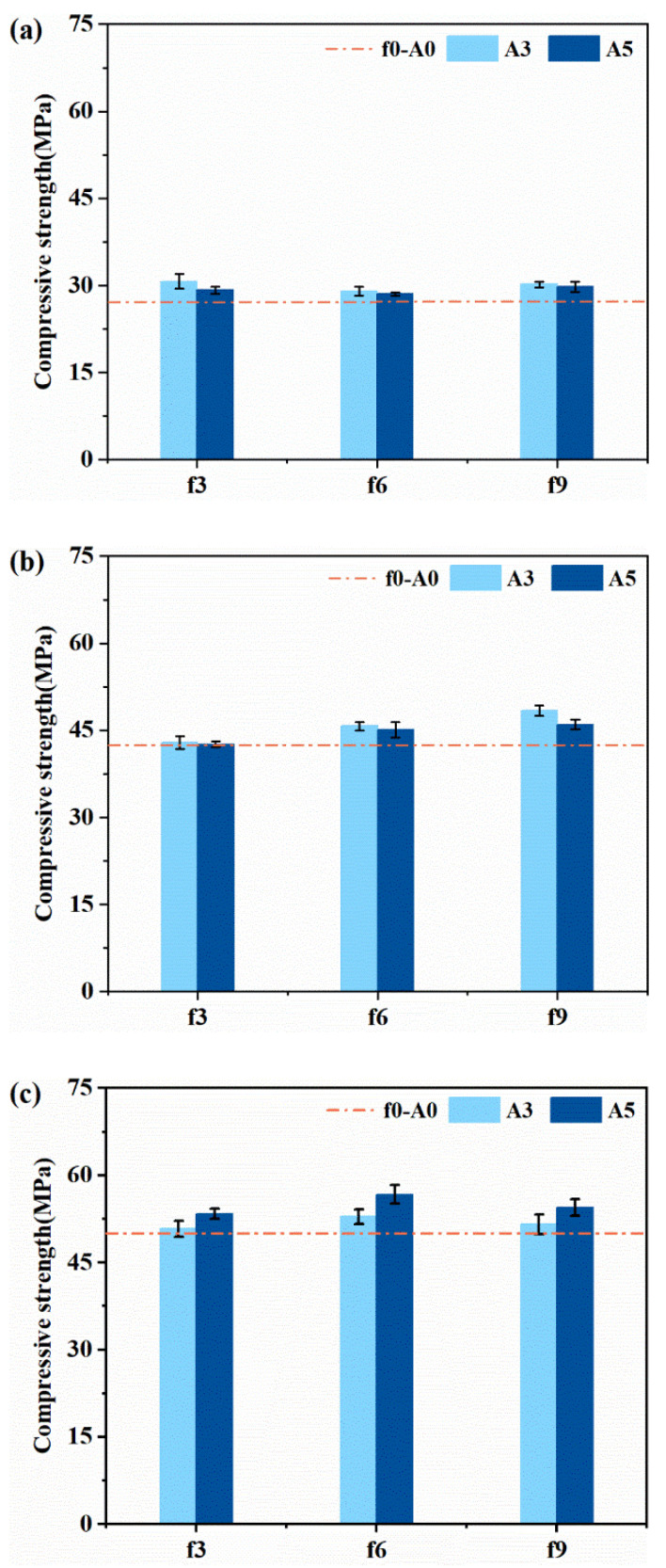
Compressive strength of plain MPC mortars at: (**a**) 1 h; (**b**) 12 h; (**c**) 3 days.

**Figure 8:**
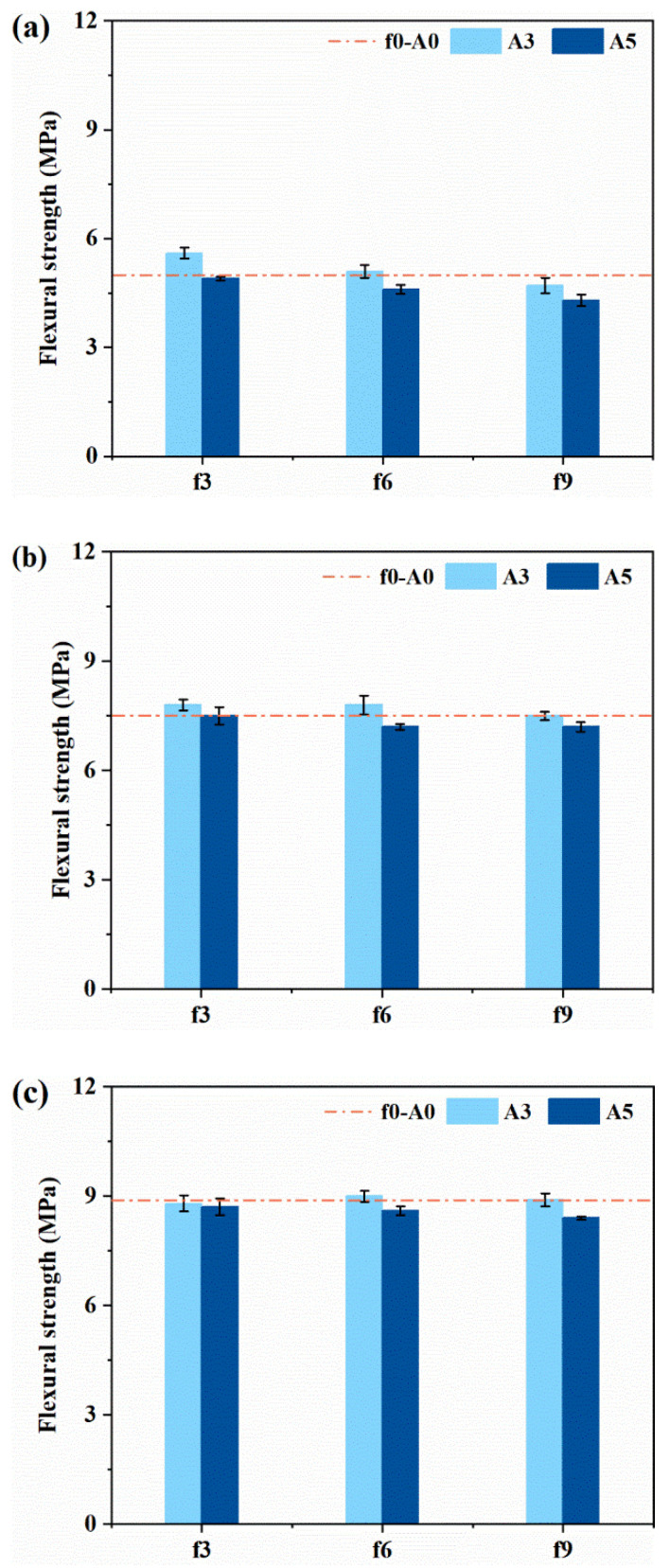
Flexural strength of plain MPC mortars at: (**a**) 1 h; (**b**) 12 h; (**c**) 3 days.

The development of compressive and flexural strength of plain MPC mortars at 1 h, 12 h and 3 days are presented in Figure 7 and Figure 8. For the specimen without vibration (f0-A0), the compressive strength was 27.2 MPa at 1 h, 42.5 MPa at 12 h and 50.0 MPa at 3 days. This indicates that 85% strength of the MPC after 3 days was achieved during the first 12 h. The high early strength of MPC should enable traffic to be opened as soon as possible when applied to pavement and bridge repairing [31].

As can be observed from Figure 7, all the vibrated specimens for all the determined ages exhibited an increased strength when compared with the reference, while the impact degree was dependent on testing age. Increasing the vibration amplitude at an early stage could be detrimental to the mechanical properties. Specimens that vibrated with an amplitude of 3 mm show a higher compressive strength during the first 12 h than that with an amplitude of 5 mm. However, the compressive strength was improved more obviously by a larger vibration amplitude at 3 days. Specimens vibrating under the frequency of 6 Hz showed a higher compressive strength than those vibrated with the frequency of 3 Hz and 9 Hz, after 3 days of curing. Due to easier upwards movement of bubbles produced by the acid–base reaction between MgO and NH_4_H_2_PO_4_, a denser matrix could be achieved by applying appropriate vibration.

The flexural strengths of plain MPC mortars at different ages are shown in Figure 8. It is worth noting that there was a tendency of reducing flexural strength for all the determined ages when a high frequency or amplitude was applied. This could be due to the fact that the flexural strength was more sensitive to internal cracks and defects caused by a high frequency or amplitude vibration [32]. However, the effect of coupled vibration on the generation and development of internal cracks was limited by the continuous formation of struvite crystal, which is the main hydration product of MPC. It can be seen that the effect of vibration on the 3-day flexural strength of MPC was insignificant.

### 3.2. Effect of Coupled Vibration on the Mechanical Strength of Fiber-Reinforced Magnesium Phosphate Cement (FR-MPC) Composites

**Figure 9:**
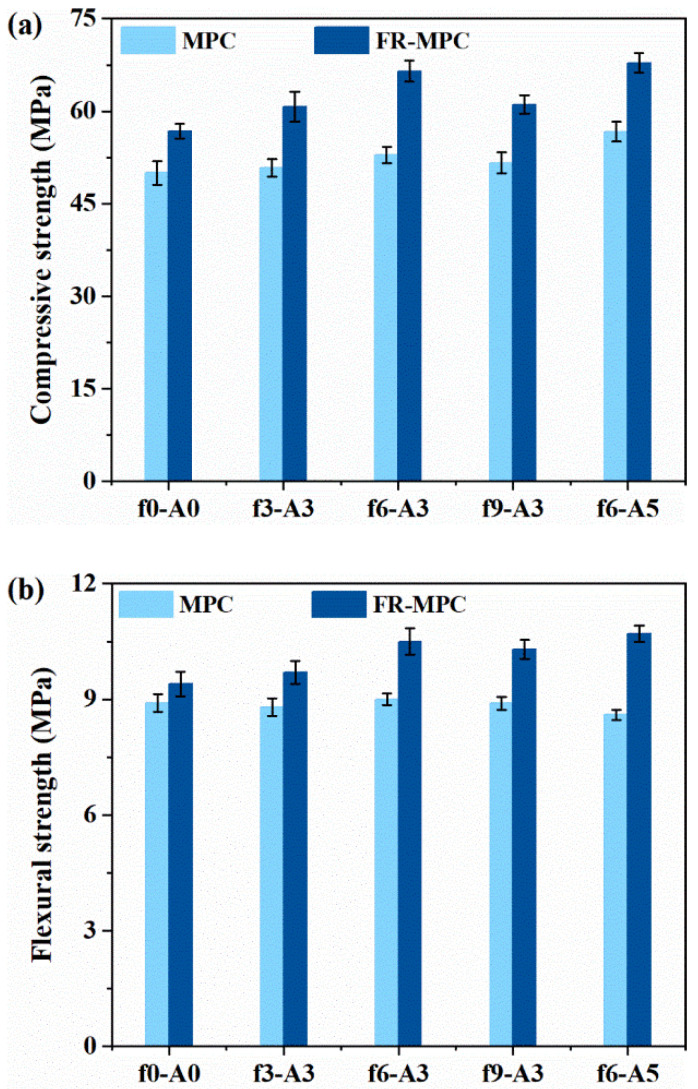
Mechanical strength of FR-MPC composites: (**a**) compressive strength; (**b**) flexural strength.

The effect of coupled vibration on the compressive strength and flexural strength of FR-MPC composites at 3 days is displayed in Figure 9. Plain MPC mortars were set as the control group. In general, the mechanical strength of MPC was slightly improved by the inclusion of micro-steel fibers. The results show that the variation trend of compressive strength of FR-MPC with vibration was similar to that of MPC. Moreover, the positive effect of vibration on the flexural strength was more prominent for FR-MPC than MPC. The increase in flexural strength of FR-MPC was due to the appropriate vibration enhancing the effective bond between the micro-steel fibers and the MPC matrix, as presented in Section 3.3. As observed, the compressive strength and flexural strength of FR-MPC first increased, then decreased with the increase in frequency, and varied slightly with the increase in amplitude. It was due to the good cohesion of FR-MPC and the very short setting time of MPC, that the change in vibration frequency showed a more obvious effect on the mechanical properties than on amplitude.

### 3.3. Effect of Coupled Vibration on the Fiber Pull-Out Behavior

**Figure 10:**
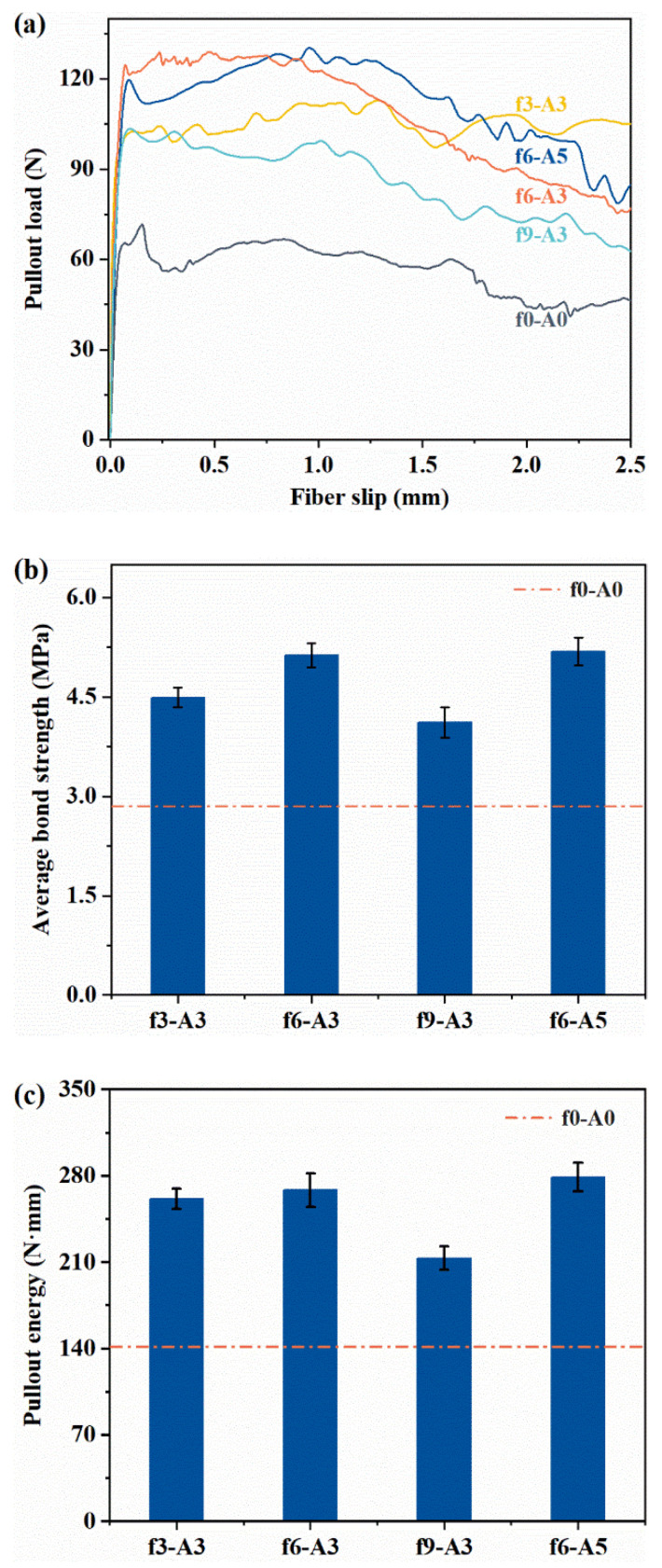
The interface bond properties between the steel fiber and MPC matrix: (**a**) pull-out load versus slip curve; (**b**) average bond strength; (**c**) pull-out energy.

To explore the effect mechanism of vibration on mechanical strength of FR-MPC composites, the pull-out load versus slip curves of the embedded micro-steel fibers in an MPC matrix is shown Figure 10a. The obtained results, including average bond strength and pull-out energy at 3 days, are summarized in Figure 10b,c. All the specimens after vibration showed a higher average bond strength and pull-out energy than the references. The average bond strength of f6-A5 was relatively close to that of f6-A3 and 80.0% higher than that of f0-A0. The f3-A3 and f9-A3 specimens were 57.5% and 44.6% higher than that of f0-A0. The pull-out energy showed a similar trend to the average bond strength. Therefore, vehicle–bridge coupled vibration was effective in enhancing the interfacial bond between the micro-steel fiber and the MPC matrix, which could be one of the critical factors influencing the above-mentioned mechanical property evaluation.

Helfet et al. [33] indicated that the flexural strength of steel fiber-reinforced cement-based materials is directly linked with the bridging action of fibers in the matrix. This bridging effect mainly depends on the bond between the micro-steel fibers and the MPC matrix [34]. When subjected to failure loading, the bridging fibers, with high modulus of elasticity across the cracks, undertake applied stress and delay micro-crack propagation to resist failure [24,35,36]. Interaction and strong bonding of the MPC matrix to the fiber after vibration could improve the load-carrying capacity of FR-MPC.

### 3.4. Effect of Coupled Vibration on the Bonding Properties of Repaired System

**Figure 11:**
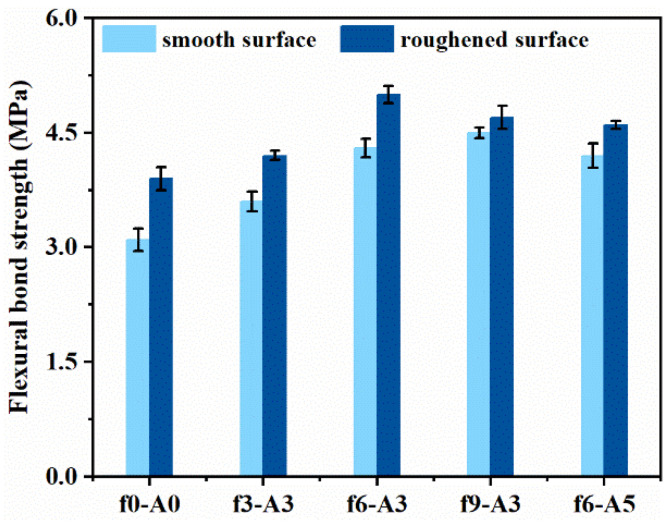
Flexural bond strength between FR-MPC and OPC substrate.

The bonding properties between repair materials and the substrate are an important factor affecting the final repaired system. Figure 11 shows the effects of vibration on the flexural bond strength between FR-MPC composites and OPC substrates of different roughness. Compared to the reference, there was a significant improvement in flexural bond strength between FR-MPC and the OPC substrate. In the case of f6-A3, the flexural bond strengths of FR-MPC with the smooth and roughened surfaces of the OPC substrates were 4.3 MPa and 5.0 MPa, respectively, corresponding to 38.7% and 28.2% improvement compared with the non-vibrated specimen, respectively. This performance boost stemming from vibration is conducive to the filling and penetration of fresh MPC paste into the pores and cracks in the interface of the OPC substrate. The flexural bond strength for the rough substrate surface was higher than that bonded to a smooth surface under the same vibration mode. The roughened surface improved the friction coefficient along with bonding area between repair materials and substrate, as reported in previous experimental studies [4].

### 3.5. Effect of Coupled Vibration on the Pore Structure of MPC Matrix

**Figure 12:**
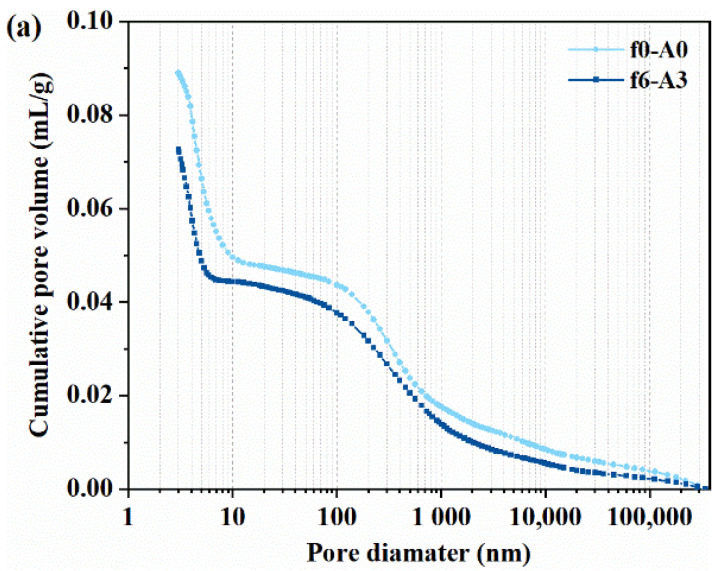

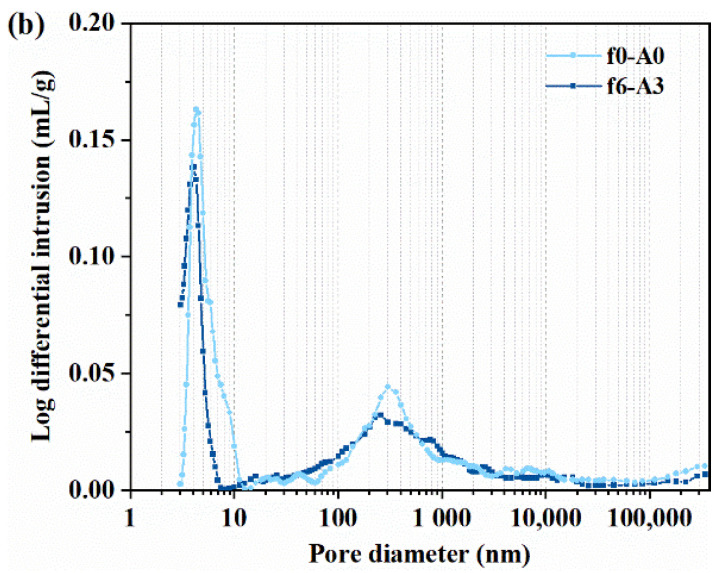
The pore distribution curve of MPC matrix: (**a**) cumulative pore volume distribution; (**b**) differential pore size distribution.

The pore structure of f0-A0 and f6-A3 obtained by the MIP test is shown in Figure 12. As shown in Figure 12a, the cumulative pore volume of f0-A0 was higher than f6-A3. The porosity of MPC decreased from 20.95% to 15.50% after vibration. From Figure 12b, the most probable diameter of MPC matrix was decreased after experiencing the coupled vibration. Moreover, the vibration was also able to reduce the volume of coarse pores. Chen et al. [21,37] divided the pore structure of MPC into two main types: coarse pores (>0.1 μm) and fine pores (≤0.1 μm). The volume percentages of coarse pores for f0-A0 and f6-A3 specimens were 50.04% and 47.66%, respectively, indicating a refined pore structure [38]. This also explains the reason for the higher compressive strength of plain MPC mortars and better interface bond properties between micro-steel fibers and the MPC matrix.

### 3.6. Effect of Coupled Vibration on the Microstructure of Bond Interface

**Figure 13:**
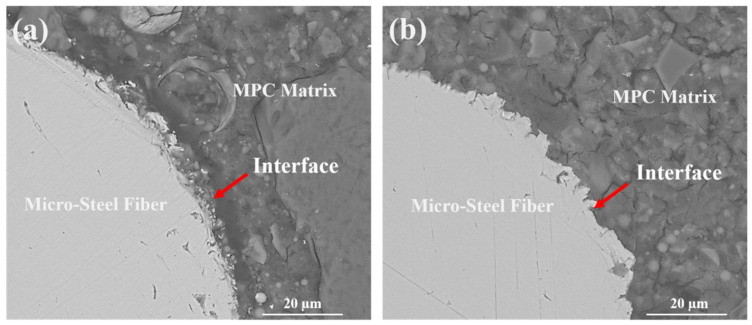
Backscattered electron images of the interface between MPC matrix and micro-steel fiber after vibration of (**a**) f0-A0; (**b**) f6-A3.

The backscattered electron (BSE) images of the interface between MPC matrix and micro-steel fiber are shown in Figure 13. In comparison with the reference specimen (f0-A0 in Figure 13a), the micro-steel fiber in the vibrated specimen was tightly embedded into the MPC matrix to form a denser interface (f6-A3 in Figure 13b). The densified interface is beneficial to the toughening effect of fiber on the matrix [39,40,41], which is in accordance with a higher load-carrying capacity of FR-MPC.

**Figure 14:**
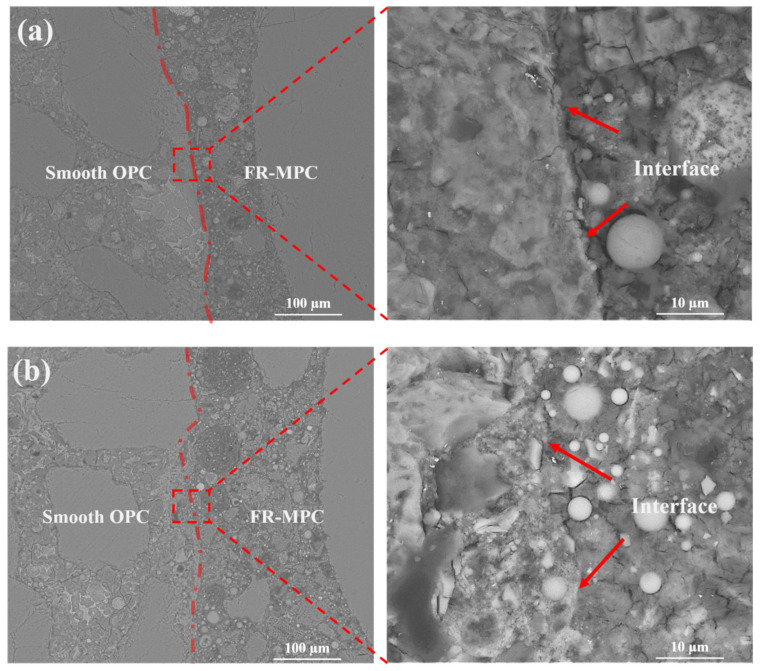
Backscattered electron images of the interface between FR-MPC composites and smooth OPC substrate after vibration of: (**a**) f0-A0; (**b**) f6-A3.

**Figure 15:**
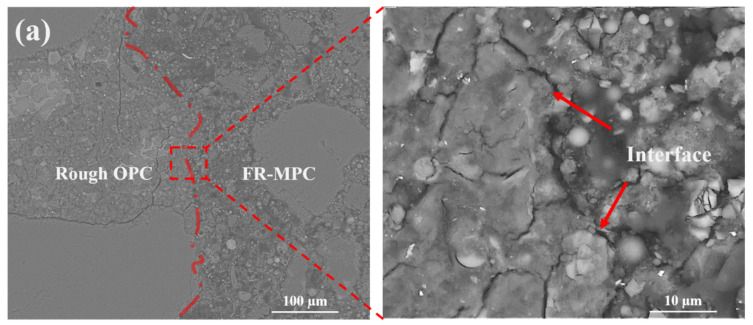

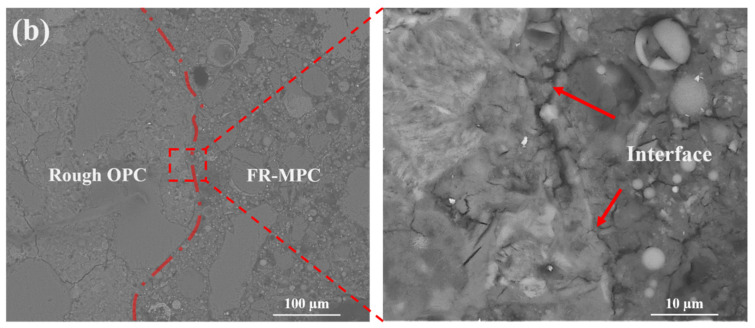
Backscattered electron images of interface between FR-MPC composites and rough OPC substrate after vibration of: (**a**) f0-A0; (**b**) f6-A3.

The BSE images of the bond interface between FR-MPC composites and OPC substrates with different roughness are shown in Figure 14 and Figure 15. Both sides of the interface have completely different structural characteristics. The continuous struvite crystal acts as a binder that can bond with the OPC substrate to provide bond strength [4,42]. Compared with the interface between FR-MPC and the smooth OPC surface shown in Figure 14, the interface between FR-MPC and the roughened surface (as shown in Figure 15) is more irregular. The irregular interface increases the contact area and provides more embedding environment for the MPC paste. Results in Figure 15a,b illustrate that, after 3 days of hydration, the microstructure in the vibrated specimen near the interface is more compact than the reference; additionally, there is no noticeable gap on the interface position. This is because vibration provides power for the MPC paste to penetrate and fill up the pores of the OPC substrate during the formation process of the interface. The microscopic characteristics of the interface are consistent with the flexural bond strength results.

## 4. Conclusions

The effect of the coupling vibration of vehicle and bridge on the mechanical properties of FR-MPC composites and the bonding properties of repaired system was investigated. The microstructure of the bonding interface between FR-MPC and OPC substrate was also determined. The following conclusions can be drawn based on the experimental results presented in this study:

(1) Vehicle–bridge coupled vibration had a positive influence on the compressive and flexural strength of FR-MPC composites, but only slightly affected the flexural strength of plain MPC mortars. The increase in mechanical capacity of FR-MPC composites was attributed to the significant improvement in interface bond properties between the micro-steel fiber and the MPC matrix.
(2) The cumulative pore volume and the volume of coarse pores in the MPC matrix can be modified by vibration, which is also beneficial for the improvement of the compressive strength of the plain MPC mortar matrix and properties of the interface bond between the fiber and the matrix.
(3) A more compact interface between FR-MPC composites and OPC substrate under vibration conditions was observed by BSE analysis. The filling and penetration of MPC paste into the pores and cracks of the OPC substrate under vibration lead to an increase in flexural bond strength between the FR-MPC and the OPC substrate. The presented work confirms that FR-MPC can be widely utilized in the case of rapid-repairing construction, even under vibration.

## Figures and Tables

**Table 1:** Chemical composition of magnesium phosphate cement (MPC) (wt.%).

Composition	SiO_2_	Al_2_O_3_	Fe_2_O_3_	CaO	MgO	SO_3_	Na_2_O	K_2_O	TiO_2_	P_2_O_5_
Content	12.58	5.90	2.74	1.73	49.49	1.51	0.98	0.41	0.51	23.4

**Table 2:** Physical properties of micro-steel fibers.

Type	Shape	Length (mm)	Diameter (mm)	Aspect Ratio (L/d)	Density (g·cm^−3^)	Tensile Strength (MPa)
1	Straight	20	0.20	100	7.85	2000
2		7	0.15	47	7.85	3000

## Data Availability

All the data is available within the manuscript.
